# Nanostructured porous silicon micropatterns as a tool for substrate-conditioned cell research

**DOI:** 10.1186/1556-276X-7-396

**Published:** 2012-07-16

**Authors:** Esther Punzón-Quijorna, Vanessa Sánchez-Vaquero, Álvaro Muñoz-Noval, M Jesus Pérez-Roldán, Raúl J Martín-Palma, Francois Rossi, Aurelio Climent-Font, Miguel Manso-Silván, J Predestinacion García Ruiz, Vicente Torres-Costa

**Affiliations:** 1 Departamento de Física Aplicada, Facultad de Ciencias, Universidad Autónoma de Madrid, Cantoblanco, Madrid, 28049, Spain; 2 Centro de Microanálisis de Materiales, Universidad Autónoma de Madrid, Cantoblanco, Madrid, 28049, Spain; 3 Departamento de Biología Molecular, Facultad de Ciencias, Universidad Autónoma de Madrid, Cantoblanco, Madrid, 28049, Spain; 4 European Commission, Joint Research Centre, Institute for Health and Consumer Protection, Ispra (VA), 21027, Italy

**Keywords:** Nanostructured porous silicon, Ion beam, Human mesenchymal stem cells, Cell adhesion, Photoluminescence, Micropattern, Cell guide

## Abstract

The localized irradiation of Si allows a precise patterning at the microscale of nanostructured materials such as porous silicon (PS). PS patterns with precisely defined geometries can be fabricated using ion stopping masks. The nanoscale textured micropatterns were used to explore their influence as microenvironments for human mesenchymal stem cells (hMSCs). In fact, the change of photoluminescence emission from PS upon aging in physiological solution suggests the intense formation of silanol surface groups, which may play a relevant role in ulterior cell adhesion. The experimental results show that hMSCs are sensitive to the surface micropatterns. In this regard, preliminary β-catenin labeling studies reveal the formation of cell to cell interaction structures, while microtubule orientation is strongly influenced by the selective adhesion conditions. Relevantly, Ki-67 assays support a proliferative state of hMSCs on such nanostructured micropatterns comparable to that of standard cell culture platforms, which reinforce the candidature of porous silicon micropatterns to become a conditioning structure for *in vitro* culture of HMSCs.

## Background

The design and fabrication of surfaces allowing the control of cell to material interactions is currently a subject of great significance, given its potential impact in the development of implantable medical devices and engineered tissues. Irradiation of materials with MeV ions has been used in the last decades to synthesize new materials and to induce luminescent or magnetic properties [1-3]. In particular, high-dose irradiation increases the resistivity of Si, inhibiting the formation of porous silicon (PS) during the subsequent anodization [4]. In this way, if a high-energy focused ion beam is electromagnetically scanned, or a wide defocused beam is shone through the appropriate mask, bidimensional patterns can be defined in a controlled way [4].

Bone progenitor cells are within a bone microenvironment replete with growth factors, nutrient, morphologic factor, and mechanical environment in an adequate combination. Cellular developments, such as fate selection, proliferation, differentiation, migration, or apoptosis, are guided by multiple surface cues that are potentially remodeled during cell culture assays [5]. Interactions between cells and the underlying surface control cellular attachment, proliferation, and activity [6]. In fact, cells respond to synthetic topographic surfaces in many different ways, which depend upon many factors including feature size and geometry, cell type, or the physicochemical properties of the particular surface [7]. In this regard, surface micropatterns have been demonstrated to be a useful tool for the control of cell behavior [8,9]. Moreover, surface nanotopography has been shown to exert influence over cell adhesion, morphology, proliferation, migration, differentiation, alignment, cytoskeleton organization, and gene expression in many cell types, including human mesenchymal stem cells (hMSCs) [10]; hMSCs are being increasingly used in therapeutic applications for bone, cartilage, and fat transplantation and repair [11]. However, success in the development of useful applications is currently limited due to the complexity of interactions that affect the differentiation and proliferation of stem cells. For many applications, a precise control of issues such as cell adhesion and migration is required. In this regard, it has been shown that mechanically induced focal adhesion amplifies anti-adipogenic pathways in mesenchymal stem cells [12].

Nanostructured PS can be described as a complex network of Si nanocrystals with large specific surface area [13]. This material shows a wide variety of interesting properties leading to applications in several fields ranging from micro- and optoelectronics to biomedical applications [14]. Regarding this particular area, it is important to note that the biocompatibility of PS strongly depends on its porosity and pore size and can be tailored as a function of the particular application [15-17]. Furthermore, PS presents reputed biocompatibility with epithelium [18], osteochondral [19], neuronal [20], and eye tissues [21], which opens the way for its use in tissue engineering applications.

## Methods

### Fabrication of Si/PS micropatterns

Surface micropatterns were engineered by defining areas of monocrystalline silicon and PS as follows: low-resistivity ohmic aluminum back contacts were deposited on the back side of boron-doped (p-type) monocrystalline Si wafers (<100 > orientation) by electron-beam evaporation, followed by thermal annealing at 400°C. Afterwards, the top surface of the silicon wafers were irradiated with He^+^ ions through 400 mesh stripe Cu micro-masks, resulting in lowered conductivity of the exposed areas of the Si wafers. The irradiation process was carried out with 1-MeV He^+^ at fluences of 5 × 10^13^ cm^−2^ using the Cockroft-Walton tandetron accelerator (High Voltage Engineering Europa B.V., Amersfoort, The Netherlands) available at CMAM [22]. The resulting micropatterns with well-defined areas of different conductivities are used to selectively grow PS regions on the surface of the Si wafers. Accordingly, after mask removal the silicon wafers were galvanostatically etched with a current density of 45 mA/cm^2^, for 60 s, in HF-to-ethanol (1:1) electrolytes under illumination following a standard PS fabrication process, resulting in Si-based spongelike structures [23,24]. The Si/PS surface patterns reproduce the geometry of the masks on the substrate; i.e., PS selectively grows in non-irradiated areas, while monocrystalline Si remains in irradiated regions. For physiological aging studies, PS was immersed in phosphate buffered saline (PBS) once for increasing times up to 1,000 min. Before characterization, the samples were rinsed in milli-Q water (Millipore Co., Billerica, MA, USA) and dried in air.

### Characterization

The morphology of the Si/PS micropatterns was studied using a Hitachi S-3000 N (Hitachi High-Tech, Minato-ku, Tokyo, Japan) scanning electron microscope (SEM) equipped with a conventional thermionic filament. The operation voltage was set at 20 KeV. Atomic force microscopy (AFM) characterization was performed by the use of an NT-MDT Solver (NT-MDT, Moscow, Russia) equipped with a Smena head. Silicon cantilevers with a force constant of 5 N/m and a first harmonic frequency of 158 KHz were used. The analysis of the images and estimations of root mean square (rms) roughness were performed using the NT-MDT software.

Photoluminescence spectra were recorded in an SLM-Aminco Bowman AB2 (SLM Instruments, Inc. Illinois, USA) spectrofluorometer. Excitation light at 250 nm was provided by a 250-W Xe lamp and was monochromatized with a 2-nm bandwidth. Emission spectra were monochromatized with a 4-nm bandwidth and detected with a photomultiplier tube operated at 800 V.

### Cell culture and immunofluorescence

Human bone marrow samples (2 to 4 ml) from healthy donors were provided by Hospital Universitario La Princesa (Madrid, Spain). Cells were collected by centrifugation on 70% Percoll gradient and seeded at 200,000 cm^−2^ in Dulbecco's modified Eagle's medium with low glucose (DMEM-LG) supplemented with 10% fetal bovine serum (FBS) (Sigma-Aldrich, Tres Cantos, Spain). The medium was replaced twice per week. To perform the cell culture, the surface micropatterns were exposed to UV light during 10 min, thoroughly washed with PBS (Tissue Culture Service, Molecular Biology Center Severo Ochoa, CSIC-UAM), placed on a 24-multiwell plate (Falcon, BD Biosciences, San Jose, CA, USA), and seeded with 15,000 cells. As control, hMSCs were plated on 0.5% gelatin-coated cover slips (bovine skin; Sigma-Aldrich Corporation, St. Louis, MO, USA). Cells were then incubated for 72 h with DMEM-LG adjusted to 10% FBS at 37°C in 5% CO_2_. After washing twice with PBS, cells were fixed in 3.7% formaldehyde in PBS for 30 min at room temperature (RT). Surface interactions between the Si/PS micropattern and hMSCs are studied, which were determined by β-catenin, α-tubulin from microtubules, and the nuclear staining [25]. Furthermore, the Ki-67 assay has been used as a reputed method for the evaluation of the proliferative state of hMSCs [26,27]. To this end, cells were permeated in 0.5% Triton X-100 in CSK buffer (100 mM NaCl, 10 mM Pipes pH 6.8, 3 mM MgCl_2_, 3 mM EGTA and 0.3 M sucrose) for 30 min at RT. Samples were blocked with 1% bovine serum albumin (BSA) in PBS for 1 h at RT. After washing, the surfaces were incubated in dark conditions for 1 h with β-catenin (1:200; Santa Cruz Biotechnology, Santa Cruz, CA, USA), 4′,6-diamidino-2-phenylindole (1:5000; Calbiochem, Merck Chemicals Ltd., Nottingham, UK), Tubulin (1:2000; Sigma-Aldrich) and Ki-67 (1:200; Clone SP6, Thermo Fisher Scientific). After blocking with 0.1% BSA in PBS, the samples were incubated in dark conditions for 45 min with secondary antibodies: Alexa 555 donkey anti-mouse (1:500; Invitrogen, Life Technologies, Carlsbad, CA, USA) and Alexa 488 donkey anti-rabbit (1:500, Invitrogen). After incubation, the surfaces were washed, dehydrated with absolute ethanol (Panreac Química S.A., Castellar del Valles, Barcelona, Spain) and mounted with Mowiol/Dabco (Calbiochem). Cells were visualized in a fluorescence inverted microscope (Olympus IX81, Olympus Corporation, Shinjuku, Tokyo, Japan) coupled to a CCD color camera.

## Results and discussion

Silicon-based micropatterns textured at the nanoscale were fabricated following the fabrication procedure described in the ‘Fabrication of Si/PS micropatterns’ subsection. It is well known that cell growth, division, and migration are highly dependent on their culture substrate. Accordingly, factors such as surface chemistry and roughness at the micro- and nanoscale determine wettability and consequently influence the biological reactions at biomaterial surfaces [28]. In this regard, these key factors, and how cell respond to them, are studied and discussed for the Si/PS micropatterns in the following paragraphs.

### Structure and properties of the Si/PS micropatterns

**Figure 1:**
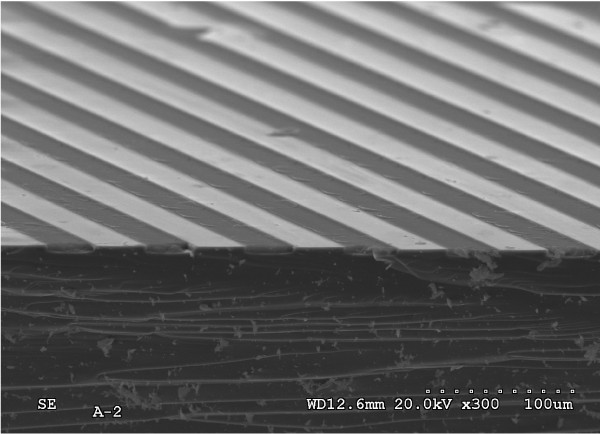
Cross-sectional SEM image of a Si/PS micropattern showing alternating Si and PS stripes.

**Figure 2:**
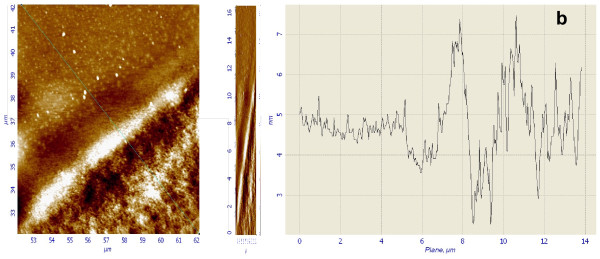
**AFM image and topography plot of the nanostructure of the Si/PS micropatterns.** ( **a**) 10 × 10 μm^2^ AFM image from a Si/PS boundary and (**b**) plot of the topographic profile from the diagonal line in (a).

Figure 1 shows a SEM micrograph showing a characteristic Si/PS surface micropattern, from which alternating Si and PS micrometric stripes can be observed. In order to get an insight into the nanotopography of the patterns, AFM images from the Si/PS transition areas were acquired, as shown in Figure 2a. The experimental results show that these boundary areas typically present a smooth topographic irregularity with total step height below 10 nm. It is observed that PS areas present their typical spongiform structure, while Si areas reflect only a slightly increased surface roughness. Data analysis showed that for scanning areas of 2 × 2 μm^2^, the surface rms roughness of the PS areas was found to be 1.1 nm, much larger than the rms roughness measured for the silicon areas which was found to be 0.2 nm. The topography plot presented in Figure 2 b clearly illustrates the previously mentioned contrast in surface nanotopography.

**Figure 3:**
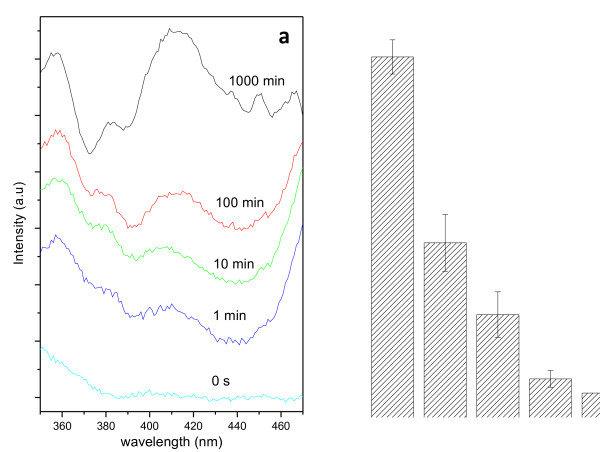
**Aging studies of PS in relevant conditions for cell culture.** Photoluminescence spectra in the 250- to 480-nm range for fresh PS and PS aged in PBS for 1, 10, 100, and 1,000 min.

Aging of PS in physiological medium was studied in order to elucidate the physicochemical changes suffered by PS surfaces under simulated saline culture conditions. Aging studies were carried out by immersion of PS layers in PBS once for times up to 1,000 min. After immersion for a given time in PBS, photoluminescence from PS was measured, as shown in Figure 3. These spectra indicate that photoluminescence intensity in the blue range increases progressively upon exposure to PBS up to 1,000 min (i.e., up to typical cell culture periods) as illustrated in Figure 3 in agreement with previously described aging in alternative conditions [29]. This indicates increased oxidation of the PS with a high density of silanol Si-OH groups, which may be responsible of a readily observed increased hydrophilicity [30,31].

Accordingly, the definition of Si/PS surface micropatterns results in simultaneous chemical and nanotopographic contrasts whose use for the control of cell adhesion will be subsequently discussed.

### Response of human mesenchymal stem cells to Si/PS micropatterns

**Figure 4:**
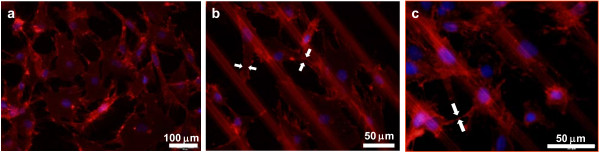
**hMSCs observed by fluorescence microscopy with nuclear and β-catenin staining.** After culture on ( **a**) gelatin covered glass controls; ( **b** and **c**) 40-μm Si/20-μm PS micropatterns at different magnifications.

Adhesion of hMSCs cultured on Si/PS surface micropatterns was studied. β-Catenin structures were stained in parallel series of cultured hMSCs in view of their mediation role between transcription regulation and transmembrane proteins associated to cell adhesion complexes and cell to cell communication. The fluorescence microscopy results indicate that the presence of a surface contrast induces a clear polarization of the hMSCs, which exhibit a remarkable elongated shape with respect to the striated cells cultured in gelatin control surfaces (Figure 4a,b for control and Si/PSi patterned surfaces, respectively). Furthermore, with the particular dimensions of 40-μm-wide Si stripes and 20-μm-wide PS stripes, the hMSCs are preferentially located on PS, as derived from the position of the nuclei (Figure 4c). Although dominantly standing on the surface of PS, hMSCs occasionally assemble on the surface of Si (see several cases in Figure 4b,c), most probably as an issue of cell to cell interactions in concurrence with cell to substrate interactions.

With respect to the structure of the β-catenin skeleton (red staining), hMSCs cultured on control substrates present diffused intensity within the nuclear perimeter and increased intensity spots on the peripheral areas (Figure 4a). Such behavior is noticeably altered for hMSCs cultured on 40-μm Si/20-μm PS micropatterns. Only cells with nuclei standing on Si areas exhibit a similar morphology. Otherwise, (for hMSCs whose nucleus stands on PSi) β-catenin addresses preferentially onto PS areas (Figure 4b,c). Note the characteristic de-wetting patterns of β-catenin cytoskeleton on Si areas (faced arrows, Figure 4b) and their convergence to PS regions. Particular β-catenin contacts between hMSCs in neighboring stripes were also observed suggesting the development of cell to cell communication structures (Figure 4c, arrows).

**Figure 5:**
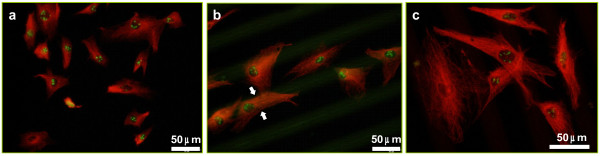
**hMSCs observed by fluorescence microscopy (Ki67 positive nuclei, tubulin stained microtubules).** After culture on ( **a**) gelatin covered glass controls; ( **b** and **c**) 40-μm Si/20-μm PS micropatterns at different magnifications.

In order to get an insight into cytoskeleton proteins with direct linking to the nuclei, a new series of samples was studied by tubulin staining and compared with the response observed on controls (Figure 5a). For hMSCs onto 40-μm Si/20-μm PS micropatterns (Figure 5b,c), the general morphology of the microtubules suggests a comparable state of the cells not differing much from that observed on controls. It is patent however that the tubulin is affected by the general substrate-induced polarization process and microtubules dominantly follow the stripe orientation (see constriction indicated by white arrows on Figure 5b). The hMSCs used for this experiment were the subject of a simultaneous proliferation study using Ki-67 assay. The proliferative rate identified by green dotted nuclei was close to 100% in both control samples and for 40-μm Si/20-μm PS micropatterns as identified from representative sets (four images) of low magnification fields.

## Conclusions

Surface micropatterns composed of alternating silicon and nanostructured porous silicon stripes have been used to study the surface distribution and shape of the human skeletal progenitor cells. The Si-based surface micropatterns textured at the nanoscale were fabricated by a combination of ion beam irradiation and subsequent electrochemical etch. The aging of the PS substrate in physiological conditions may play a relevant role in the creation of a wettability contrast with respect to Si areas. These chemically and morphologically patterned surfaces have been used to study cell adhesion, and hMSCs cultured on them exhibit a clear polarization with respect to the designed stripes. With respect to the origin of this polarization effects, the photoluminescence spectra from PS aged in physiological saline conditions strongly suggest that the OH groups induced could be responsible of an increased surface wettability. However, an effect derived from the porous nature (i.e., allowing molecular permeability at the adhering site of the cell) or a slight influence of the nanometric transition step in the Si/PS transitions cannot be discarded. Although this polarization effect remains to be quantified (dependence on time of culture, on levels of cell to cell competing condition, etc.), these preliminary assays provide clear statistics on hMSC proliferation at comparable rates with respect to the culture on standard substrates. Such Si/PS substrates become thus ideal candidates for the study of alternative microenvironment conditions to the standard flat two-dimensional plates used for hMSC differentiation studies.

## Competing interests

The authors declare that they have no competing interests.

## Authors’ contributions

The experiments presented in this work were designed by VTC, MMS, RJMP, ACF, and JPGR. The ion implantation was carried out by EPQ and ACF. VTC and EPQ performed the electrochemical etching treatment. AMN and MMS participated in performing the aging studies by PBS immersion and photoluminescence characterization. MJPR and FR studied the topography by AFM. VSV and JPGR performed the cell culture. The manuscript was written by EPQ, RJMP, VTC, MMS, JPGR, and the last version was revised by all the authors (EPQ, VSV, AMN, MJPR, RJMP, FR, ACF, MMS, JPGR, VTC). All authors read and approved the final manuscript.
